# USP18 Is Associated with PD-L1 Antitumor Immunity and Improved Prognosis in Colorectal Cancer

**DOI:** 10.3390/biom14091191

**Published:** 2024-09-21

**Authors:** Cili Jifu, Linxia Lu, Jiaxin Ding, Mengjun Lv, Jun Xia, Jingtao Wang, Peijun Wang

**Affiliations:** 1 College of Basic Medicine, Jiamusi University, Jiamusi 154007, China; 208053016@stu.jmsu.edu.cn (C.J.); lulinxia@stu.jmsu.edu.cn (L.L.);; 2 College of Public Health, Jiamusi University, Jiamusi 154007, China

**Keywords:** USP18, colorectal cancer, tumor immune microenvironment, immune checkpoint genes, PD-L1, anticancer immunity

## Abstract

Background: Compared with conventional chemotherapy and targeted therapy, immunotherapy has improved the treatment outlook for a variety of solid tumors, including lung cancer, colorectal cancer (CRC), and melanoma. However, it is effective only in certain patients, necessitating the search for alternative strategies to targeted immunotherapy. The deubiquitinating enzyme USP18 is known to play an important role in various aspects of the immune response, but its role in tumor immunity in CRC remains unclear. Methods: In this study, multiple online datasets were used to systematically analyze the expression, prognosis, and immunomodulatory role of USP18 in CRC. The effect of USP18 on CRC was assessed via shRNA-mediated knockdown of USP18 expression in combination with CCK-8 and colony formation assays. Finally, molecular docking analysis of USP18/ISG15 and programmed death-ligand 1 (PD-L1) was performed via HDOCK, and an ELISA was used to verify the potential of USP18 to regulate PD-L1. Results: Our study revealed that USP18 expression was significantly elevated in CRC patients and closely related to clinicopathological characteristics. The experimental data indicated that silencing USP18 significantly promoted the proliferation and population-dependent growth of CRC cells. In addition, high USP18 expression was positively correlated with the CRC survival rate and closely associated with tumor-infiltrating CD8+ T cells and natural killer (NK) cells. Interestingly, USP18 was correlated with the expression of various chemokines and immune checkpoint genes. The results of molecular docking simulations suggest that USP18 may act as a novel regulator of PD-L1 and that its deficiency may potentiate the antitumor immune response to PD-L1 blockade immunotherapy in CRC. Conclusions: In summary, USP18 shows great promise for research and clinical application as a potential target for CRC immunotherapy.

## 1. Introduction

Globally, colorectal cancer (CRC) is one of the deadliest cancers, accounting for 11% of all cancer diagnoses, with more than 1.85 million cases and 850,000 deaths per year. Therefore, the study of its treatment is highly clinically and publicly important [1,2]. In addition to radiochemotherapy and surgery, the use of immune checkpoint inhibitors (ICIs) in immunotherapy in recent years has provided a new treatment strategy for patients with advanced or refractory CRC, especially targeted therapy involving the important immune checkpoint proteins (ICPs) programmed death protein-1 (PD-1) and its ligand programmed death-ligand 1 (PD-L1). However, only a small proportion of CRC patients with defective mismatch repair or high microsatellite instability (dMMR/MSI-H) can benefit from the use of ICIs. Further studies have shown that in a cohort of 28 patients with MSI-high tumors, the response rate was 50%, and the disease control rate was 89% [3,4]. Therefore, more in-depth studies on the biology, microenvironment, and immunotherapy of CRC are needed to identify promising strategies to increase the clinical efficacy of antitumour immunotherapy.

In the context of immunotherapy, PD-1 serves as an immune checkpoint molecule that is expressed primarily on the surface of T cells [5,6,7]. It binds to its ligand PD-L1/2 (programmed death-Ligand 1/2), which is expressed on antigen-presenting cells and cancer cells [6,8]. Activation of the PD-1/PD-L1 pathway inhibits T cell activity and reduces cytokine production, thereby suppressing the immune response and promoting tumor growth and spread. This new anticancer approach, which aims to fully utilize the body’s own immune system to resist and fight cancer and kill cancer cells by blocking the PD-1/PD-L1 signaling pathway, has successively defeated nearly 20 major solid tumors, including colorectal cancer, substantially improved the survival of patients with advanced tumors, and has become a ‘special effect’ drug for patients with tumors [9,10]. Because PD-L1 protein expression changes dynamically during tumor progression, immunotherapy is affected by a variety of factors, including the PD-L1 expression level [11], mutational load [12], immune cell infiltration and function [13], immune checkpoint coexpression [14], and the gut microbiota [15]. Therefore, more in-depth studies on the regulatory mechanisms of PD-L1 are needed to maximize the advantages of PD-L1 monotherapy [16].

Several studies have demonstrated that deubiquitinating enzymes (DUBs), such as USP5, USP8, OTUB1, and USP12, play pivotal roles in activating immune cells and regulating the expression of molecules such as PD-L1. This regulation helps manage the immune escape strategies of tumor cells, thereby creating a complex network that influences tumor biological behavior and therapeutic responses [17,18,19,20]. Ubiquitin-specific peptidase 18 (USP18), a member of the DUB family, was initially identified for its role in de-ISGylation, which involves removing interferon-stimulated gene 15 (ISG15) from substrate proteins [21]. ISG15 has been shown to enhance antitumor immunity by increasing the K48-linked ubiquitin chain modification of PD-L1, enhancing its ubiquitination, thereby increasing the rate of degradation by the proteasomal pathway targeted by glycosylated PD-L1 and promoting the infiltration of cytotoxic T cells in the TME [22,23]. Additionally, USP18 serves as a strong inhibitory regulator of type I interferon signaling by interfering with the interaction between STAT2 and the type I interferon receptor subunit R2 and by inhibiting the activation of JAK kinase [21,24]. Reports have also linked loss of function or mutations in the IFN signaling pathway to immune escape from drug-resistant CRC [25,26,27]. However, the clinical relevance of USP18 in human CRC and its potential mechanisms in tumor immunotherapy remain to be fully elucidated.

In this study, we investigated the expression patterns of USP18 in both CRC patient samples and cell lines, as well as its role in augmenting antitumor immunity in the context of CRC. Moreover, our results suggest that USP18 could be a novel target for PD-L1, impacting PD-L1 expression levels. We emphasize the importance of USP18 in CRC progression and investigate its potential role in enhancing the therapeutic efficacy of ICIs.

## 2. Materials and Methods

### 2.1. USP18 Expression Analysis

The USP18 mRNA expression levels across 33 types of tumors were obtained from the TIMER 2.0 online database (http://timer.comp-genomics.org, accessed on 22 February 2024). The expression of USP18 in CRC was validated via the GEPIA database (http://gepia.cancer-pku.cn/, accessed on 25 February 2024) and the GSE87211 dataset from the Gene Expression Omnibus (GEO) database (http://www.ncbi.nlm.nih.gov/geo/, accessed on 17 March 2024). Additionally, the correlations between USP18 expression and various clinicopathological parameters (such as age, gender, histological type, and lymph node metastasis status) in CRC patients were analyzed via the UALCAN online database (https://ualcan.path.uab.edu/analysis.html, accessed on 10 November 2023).

### 2.2. Kaplan-Meier (KM) Plotter Database Analysis

We examined overall survival (OS) and recurrence-free survival (RFS) in CRC patients grouped into high and low USP18 expression categories via the KM Plotter (https://kmplot.com/analysis/, accessed on 13 April 2024) [28]. The expression groups were determined with the “automatically select the best cutoff value” feature. The significance of the differences in survival between the groups was assessed via the log-rank test, from which the p value was derived.

### 2.3. Cell Culture

Human CRC cell lines (HT29, SW620, and SW480) were obtained from the Cell Bank of the Chinese Academy of Sciences (Shanghai, China). Normal human colonic epithelial cells (NCM460) were obtained from Cyagen Biosciences (Guangzhou, China). SW480 cells were maintained in Leibovitz’s L-15 medium supplemented with 10% fetal bovine serum (FBS) and cultured without CO_2_. Conversely, the HT29 and SW620 cell lines were grown in Dulbecco’s modified Eagle’s medium (DMEM) supplemented with 10% FBS and incubated at 37 °C in a controlled environment containing 5% CO_2_. All culture media were supplemented with 1% penicillin/streptomycin. The authenticity of all the cell lines was confirmed via short tandem repeat (STR) analysis.

### 2.4. Cell Transfection

Lentiviral vectors carrying shRNAs against human USP18 were used to knock down USP18 expression. The sequences used were as follows: shUSP18#a (930–951): 5′-CRCGATTCTCCATCAGGAATTC-3′; shUSP18#b (1200–1221): 5′-CCAGTGTACCTACGGAAATCC-3′; shUSP18#c (1727–1748): 5′-CCAACATTAATTCCATATGAA-3′; nonspecific scrambled shRNAs were transfected as the control, labeled shRNA#NC (‘5-ACAGAACRCGATTGTTGATC-3’). All vectors were supplied by GeneCopoeia, Inc., Rockville, MD, USA, catalog numbers HSH117922-LVRU6GP-c and CSHCTR001-3-LVRU6GP, respectively. Following the manufacturer’s guidelines, the cells were transfected with lentiviral vectors. Three days post-transfection, the cells were selected with puromycin. The cells were subsequently harvested for experiments.

### 2.5. qRT-PCR

We extracted total RNA from the cell samples via TRIzol reagent (Solarbio, Beijing, China). We used a 1 μg RNA sample to synthesize cDNA via a reverse transcription kit (Beyotime Biotechnology, Shanghai, China, cat. no. D7168M). The reactions were programmed for 40 cycles at 95 °C for 10 s and 60 °C for 30 s. The ΔCT approach was used to compare gene expression results quantitatively, with β-actin serving as the endogenous control. The primer sequences for all the genes are shown in Supplementary Materials Table S1.

### 2.6. Western Blotting

The cells were lysed using RIPA lysis buffer (Boster Biological, Wuhan, China) and subsequently centrifuged at 15,000× *g* for 15 min. The supernatant was mixed with 1× loading buffer and boiled for 10 min. The samples were then subjected to 10% SDS-PAGE and transferred onto a PVDF membrane. To block nonspecific protein binding, the membranes were incubated with 5% nonfat milk for 1.5 h. The membranes were subsequently incubated overnight at 4 °C with the following primary antibodies: rabbit anti-USP18 (D4E7; Cell Signaling, Frankfurt, Germany; 1:1000 dilution) and β-actin (1:1000 dilution). After washing, the membranes were incubated with suitable secondary antibodies for one h. The protein bands were detected via a chemiluminescence imaging system (Amersham Imager 600, Amersham Biosciences, Piscataway, NJ, USA) and enhanced chemiluminescence (ECL) detection reagent (Yeasen, Shanghai, China). The intensities of the bands were quantified using ImageJ software (version 2.0.0).

### 2.7. Cell Proliferation Assay

Using a CCK-8 assay (Boster Biological, Wuhan, China), we assessed the proliferation of CRC cells following USP18 knockdown. The cells were seeded in 96-well plates at a density of 4 × 10^3^ cells per well and incubated at 37 °C and 5% CO_2_ for 0, 24, 48, 72, or 96 h. We added 10 μL of CCK-8 reagent to each well and incubated the cells at 37 °C for 1 h. The absorbance was then measured at 450 nm.

For the cloning experiments, the cells were seeded in a 6-well plate at an appropriate density for even distribution. Each well was supplemented with culture medium to maintain cell nutrition. The plate was incubated at 37 °C with 5% CO_2_ for 14 days, and the medium was changed every 2–3 days. After 14 days, the cells were gently washed with PBS and stained with crystal violet for 15–20 min. Excess stain was removed with tap water or PBS until clear. The cell colonies were observed and photographed under a microscope. Colony number and size were analyzed from the photos, and the data were recorded for further analysis.

### 2.8. Data Acquisition and Analysis

The TCGA database (https://portal.gdc.cancer.gov/, accessed on 10 November 2023) was used to collect RNA-seq data and clinical information from 698 CRC samples, including 51 normal colorectal tissues and 647 colorectal cancer tissues. We used the median expression value of USP18 in each sample as the cutoff to divide all samples into high and low USP18 expression groups. The R package “limma” was used for differential expression analysis, with a log fold change >1.0 and an adjusted *p* value < 0.05 as the thresholds to identify DEGs between the high and low USP18 expression groups. The R package “Enhanced Volcano” was used to plot the volcano map.

### 2.9. Gene Set Enrichment Analysis (GSEA)

By analyzing the TCGA dataset using GSEA v4.0.3, we assessed trends in the enrichment of predefined gene sets in relation to phenotypic correlations and explored the potential impact of these gene sets on phenotypes [29]. Specifically, we divided the samples into two groups with high and low USP18 expression and performed 1000 random permutations against each group to assess significant differences in gene expression. In the GSEA, phenotype labels and gene expression matrices were entered, and the enrichment of USP18-related pathways in each phenotype was ranked via normalized enrichment scores (NESs) and *p* values. Then, core enriched genes were selected to further understand the interactions between these core genes. We used the STRING database for protein-protein interaction (PPI) analysis of the selected genes. The PPI network was visualized by Cytoscape software 3.7.2, and the topology of the network was analyzed to identify key nodes and important interaction modules.

### 2.10. Functional Enrichment Analysis of the Immune Microenvironment

We utilized the CIBERSORT algorithm [30] for functional enrichment analysis of the immune microenvironment to investigate immune cell infiltration. Additionally, we employed the ESTIMATE algorithm to calculate stromal, immune, and ESTIMATE scores, further elucidating the cellular composition of the TME. For this analysis, we used the immunomodulator module of the TISIDB database to examine various types of immune cells, including activated CD8+ T cells, macrophages, and natural killer T (NKT) cells. The correlation between USP18 mRNA expression and immune cell abundance was assessed by calculating Pearson correlation coefficients (R) and *p* values and was visualized via scatter plots.

### 2.11. Single-Cell Sequencing Analysis

The Tumor Immune Single-cell Hub 2 (TISCH2) is a comprehensive single-cell RNA sequencing (scRNA-seq) database dedicated to analyzing the tumor microenvironment (TME). Within the “Datasets” module, we visualized the expression levels of USP18 at the single-cell level across three CRC datasets.

### 2.12. Protein–Protein Docking

PD-L1 (PDB ID: 4Z18) and ISG15 (PDB ID: 1Z2M) were obtained from the PDB database, whereas USP18 was obtained from the Alphafold Protein Structure Database [31]. The structures of all the proteins were prepared via a standard protein processing protocol in Discovery Studio 2019 software (v2019 client; Dassault Systèmes BIOVIA, San Diego, CA, USA). This protocol included the removal of water molecules and the addition of hydrogen atoms. We used the HDOCK server for molecular docking, which employs a global search strategy based on fast Fourier transform (FFT) to broadly sample potential binding modes of the proteins. Subsequently, it reevaluates all the collected binding modes through an iterative knowledge-based scoring function [32]. The binding affinity and dissociation constants of the protein-peptide complexes were then predicted using the online server PRODIGY (https://wenmr.science.uu.nl/prodigy/, accessed on 26 April 2024) [33].

### 2.13. Enzyme-Linked Immunosorbent Assay (ELISA)

We determined the levels of ISG15 and PD-L1 in cell culture medium supernatants using specific ELISA kits. ISG15 levels were measured with an ISG15 ELISA Kit (E-EL-H0203, Elabscience, Wuhan, China), and PD-L1 levels were assessed using a PD-L1 ELISA Kit (JL19323, Jianglaibio, Shanghai, China).

### 2.14. Statistical Analysis

Statistical analyses were performed using R and GraphPad Prism 8.0 software. The data are presented as the means ± standard deviations (SDs), with all the experiments including at least three biological replicates. When two groups of data were compared, two-tailed paired *t*-tests, unpaired *t*-tests, or Welch’s *t*-tests were used. For multigroup comparisons of a single variable, one-way analysis of variance (ANOVA) was used, followed by Tukey post hoc tests. Comparisons with *p* values < 0.05 were considered statistically significant, with * *p* < 0.05, ** *p* < 0.01, and *** *p* < 0.001.

## 3. Results

### 3.1. USP18 Is Highly Expressed in CRC Patients

**Figure 1:**
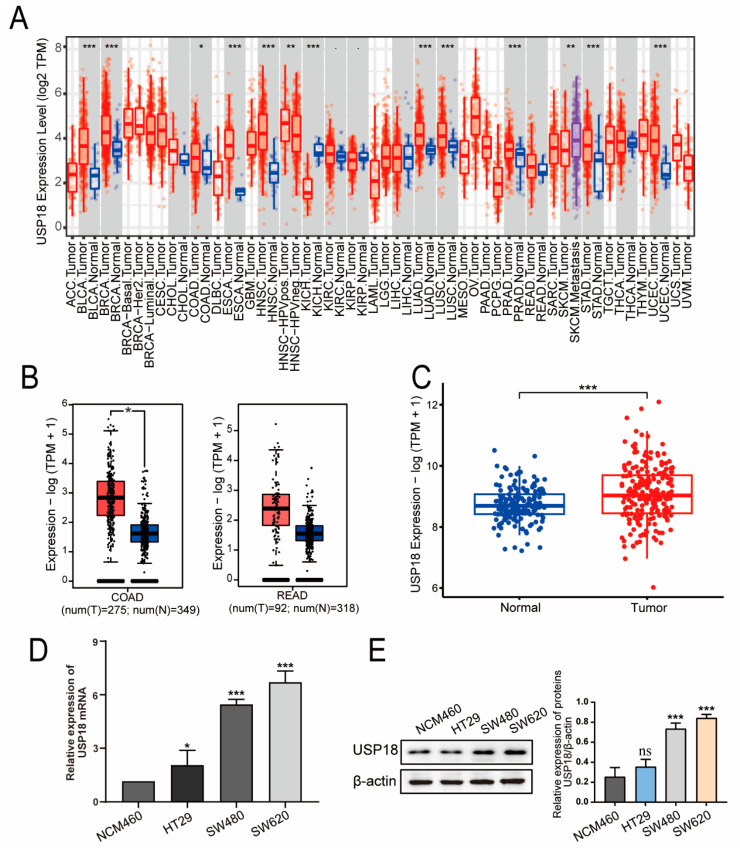
USP18 expression in colorectal cancer (CRC) samples from different databases. (**A**) The expression levels of USP18 in different cancer types in the TCGA database were analyzed via TIMER 2.0. (**B**) In the GEPIA database, USP18 was differentially expressed at different levels in colorectal adenocarcinoma (COAD) (*n* = 275), adjacent normal colon (*n* = 349), rectum adenocarcinoma (READ) (*n* = 92), and adjacent normal rectal (*n* = 318) tissues. (**C**) Differential USP18 expression in the GSE87211 dataset, with CRC tissues (*n* = 203) shown in red and adjacent normal tissues (*n* = 106) shown in blue. (**D**,**E**) Differences in the mRNA and protein levels of USP18 in NCM460, SW480, SW620, and HT29 cells were detected via qRT-PCR and Western blotting. Original blots/gels can be found at Supplementary figures. Representative data were collected from at least three independent experiments and are expressed as the means ± SD. ns, no significant difference, * *p* < 0.05, ** *p*  <  0.01, *** *p*  <  0.001.

To investigate USP18 expression in various cancers, we used TIMER 2.0 for evaluation. As depicted in Figure 1A, USP18 expression was upregulated in invasive breast carcinoma, colon cancer, head and neck squamous cell carcinoma, lung adenocarcinoma, lung squamous cell carcinoma, esophageal cancer, prostate cancer, bladder urothelial carcinoma, gastric carcinoma, and endometrial carcinoma tissues and downregulated in cutaneous melanoma and renal clear cell carcinoma tissues compared with adjacent noncancerous tissues. Further verification via the GEPIA database revealed elevated USP18 expression in colorectal adenocarcinoma (COAD) and rectum adenocarcinoma (READ) tissues relative to adjacent normal tissues, as shown in Figure 1B. Additionally, analysis of the GSE87211 dataset revealed significantly greater USP18 expression in CRC tissues than in adjacent tissues, as illustrated in Figure 1C. To validate the expression level of USP18 in CRC cells, we selected CRC cell lines representing different stages of disease progression for our study. HT29 and SW480 cells represent the early and intermediate stages of CRC development, respectively, whereas SW620 cells represent the advanced stage of CRC development. These cell lines cover different stages of CRC, from localized growth to distant metastasis. We evaluated the mRNA and protein expression levels of USP18 in these cell lines. As shown in Figure 1D,E, the mRNA and protein levels of USP18 were significantly increased in HT29, SW480, and SW620 cells, and the difference was especially significant in SW480 and SW620 cells compared with NCM460 cells. These results suggest that USP18 expression is upregulated in CRC and may play a key regulatory role in the progression of CRC.

### 3.2. Associations of USP18 Expression with Clinicopathological Features and the Prognosis of CRC Patients

**Figure 2:**
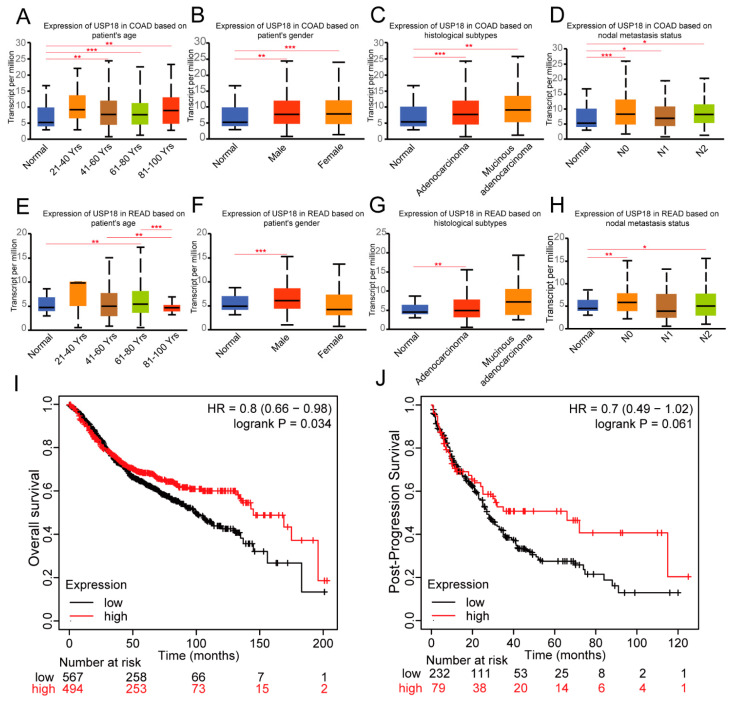
Correlations of USP18 expression with clinicopathological data and prognosis in CRC patients. (**A**) Expression of USP18 across age groups in COAD patients: normal (*n* = 41), 21–40 years (*n* = 12), 41–60 years (*n* = 90), 61–80 years (*n* = 149), and 81–100 years (*n* = 32). (**B**) Expression of USP18 by gender in COAD patients: normal controls (*n* = 41), male patients (*n* = 156), and female patients (*n* = 127). (**C**) Expression of USP18 by histological subtype in COAD patients: normal (*n* = 41), adenocarcinoma (*n* = 243), and mucinous adenocarcinoma (*n* = 37). (**D**) Expression of USP18 based on nodal metastasis status in COAD patients: normal (*n* = 41), no metastasis (N0, *n* = 166), 1–3 involved lymph nodes (N1, *n* = 70), and 4 or more involved lymph nodes (N2, *n* = 47). (**E**) Expression of USP18 across age groups in READ patients: normal (n = 10), 21–40 years (*n* = 4), 41–60 years (*n* = 53), 61–80 years (*n* = 97), and 81–100 years (*n* = 11). (**F**) Expression of USP18 by gender in READ patients: normal controls (*n* = 10), male patients (*n* = 90), and female patients (*n* = 75). (**G**) Expression of USP18 by histological subtype in READ patients: normal (*n* = 10), adenocarcinoma (*n* = 146), and mucinous adenocarcinoma (*n* = 12). (**H**) Expression of USP18 based on nodal metastasis status in READ patients: normal (*n* = 10), no metastasis (N0, *n* = 84), 1–3 involved lymph nodes (N1, *n* = 45), and 4 or more involved lymph nodes (N2, *n* = 33). (**I**) Kaplan-Meier OS analysis for CRC patients with high vs. low expression of USP18: high expression (*n* = 837) and low expression (*n* = 899). (**J**) Kaplan-Meier PFS analysis for CRC patients with high vs. low expression of USP18: high expression (*n* = 162) and low expression (*n* = 432). Overall survival (OS), progression-free survival (PFS), hazard ratio (HR). * *p* <  0.05, ** *p*  <  0.01, *** *p*  <  0.001.

To delve deeper into the influence of USP18 on CRC, we assessed the correlation between USP18 mRNA levels and a variety of clinicopathological characteristics in CRC. Patient characteristics—such as age, gender, histological classification, and lymph node metastasis status—were identified via the UALCAN database. Our first finding was that the age of the patient may be an important factor in USP18 expression, and the analysis revealed that USP18 expression was significantly different between age groups (COAD in the 61- to 80-year-olds compared with that in the control group, *p* = 0.0005966; READ in the 61- to 80-year-old group compared with the control group, *p* = 0.003042) (Figure 2A,E). Compared with those in the control group, the USP18 expression levels were elevated in all the COAD patients (Figure 2B). On the other hand, only male patients had similar and statistically significant differences in the incidence of READ (Figure 2F), which may be due to sample size limitations. In addition, the expression of USP18 was significantly different across different histologic types and lymph node metastasis statuses (normal versus interstitial/mucinous adenocarcinoma in COAD patients, *p* = 0.0001438/0.004078; normal versus adenocarcinoma in READ patients, *p* = 0.003756) (Figure 2C,G). In the context of lymph node metastasis, USP18 expression was significantly higher in early (N0 stage) COAD and READ tissues than in normal tissues (Figure 2D,H), suggesting that USP18 could be used as a biomarker for the early detection of CRC. We then evaluated the prognostic significance of USP18 in CRC patients. As shown in Figure 2I,J, compared with patients in the USP18 low-expression group, patients in the USP18 high-expressing colorectal cancer group had significantly longer overall survival (OS) (HR = 0.8, *p* = 0.034) and prolonged progression-free survival (PFS), but these differences were not statistically significant (HR = 0.7, *p* = 0.061). Overall, we found that USP18 was positively correlated with survival in CRC patients.

### 3.3. Silencing USP18 Promotes CRC Cell Proliferation and Colony Formation

**Figure 3:**
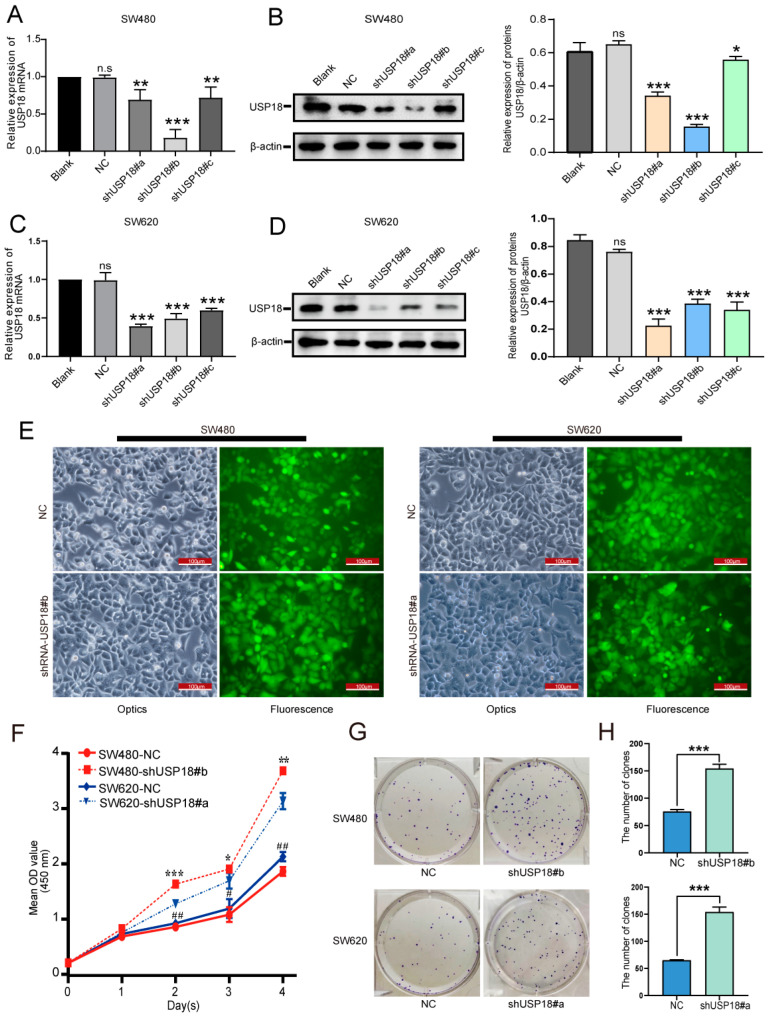
Effects of USP18 on CRC cell proliferation and colony formation. (**A**–**D**) USP18-specific shRNAs (shUSP18#a, shUSP18#b, and shUSP18#c) significantly reduced the endogenous mRNA and protein levels of USP18 in SW480/SW620 cells. (**E**) Fluorescence microscopy images showing negative control (NC) and USP18-shRNA-transfected SW480/SW620 cells at 100× magnification, with shUSP18#b in SW480 cells and shUSP18#a in SW620 cells. (**F**) Proliferation of CRC cells was assessed using the CCK-8 assay. “*” indicates SW480 cells, and “#” indicates SW620 cells. shUSP18#b was applied to SW480 cells, and shUSP18#a was applied to SW620 cells. The assay was performed with 5 replicates per group, and data were collected from at least 3 independent experiments. (**G**) Results of the colony formation assay visualized with crystal violet staining 14 days postinoculation. (**H**) Statistical analysis of colony formation efficiency among the different experimental groups. Representative data were collected from at least three independent experiments and are expressed as the means ± SD. ns, no significant difference, * *p*  <  0.05, ** *p*  <  0.01, *** *p*  <  0.001; # *p*  <  0.05, ## *p*  <  0.01.

shRNAs targeting different regions of the human USP18 gene (designated shUSP18#a, shUSP18#b, and shUSP18#c) were constructed to investigate the role of USP18 in CRC cells. Compared with the control, all three shRNAs significantly reduced the USP18 mRNA and protein levels in SW480 and SW620 cells (Figure 3A–D). The shUSP18#b and shUSP18#a groups were selected for further experiments in both cell lines. Subsequent fluorescence microscopy confirmed successful viral transfection (Figure 3E). Moreover, USP18 silencing increased the proliferation of CRC cells in a time-dependent manner (Figure 3F). Furthermore, colony formation assays indicated that USP18 silencing significantly increased the number of colonies formed by CRC cells (Figure 3G,H). Collectively, these findings demonstrate that USP18 silencing promotes the growth of CRC cells.

### 3.4. Potential Biological Functions and Pathways of USP18 in CRC

**Figure 4:**
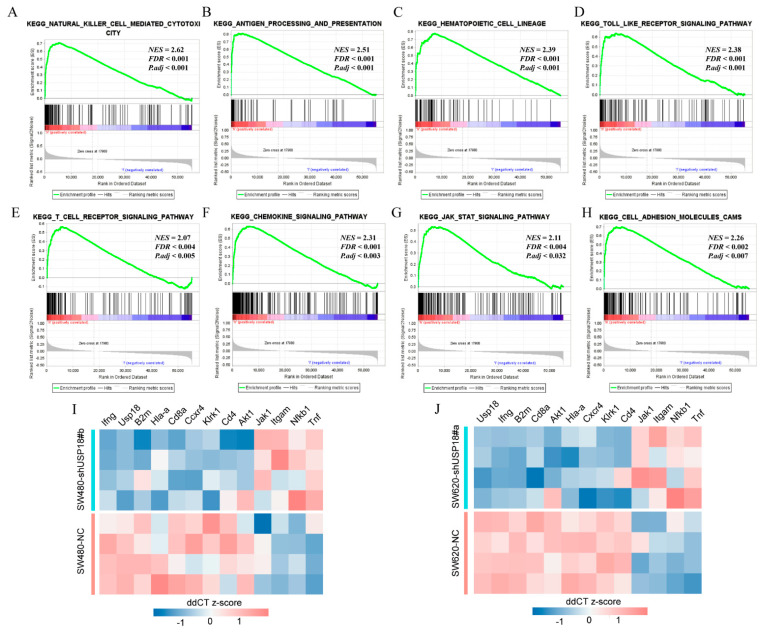
Enrichment maps from GSEA. (**A**) NK cell activity. (**B**) Antigen processing and presentation. (**C**) Hematopoietic cell lineage. (**D**) Toll-like receptor signaling. (**E**) T cell receptor signaling. (**F**) Chemokine signaling. (**G**) JAK-STAT signaling. (**H**) Cell adhesion molecules. (**I**,**J**) Changes in the relative expression of immune-related genes after USP18 knockdown (SW480-shUSP18#b and SW620-shUSP18#a) compared with negative controls (SW480-NC and SW620-NC). Gene expression levels are expressed as ddCT z-scores, *n* = 4 per group. Red indicates upregulation, and blue indicates downregulation. NES indicates the normalized enrichment score; FDR indicates the false discovery rate.

To evaluate the potential mechanisms of USP18 in CRC, we utilized GSEA and identified several significantly enriched biological pathways in the USP18 high-expression group (Figure 4A–H). These pathways included hematopoietic cell lineage, NK cell activity, the Toll-like receptor signaling pathway, antigen processing and presentation, the T cell receptor signaling pathway, the chemokine signaling pathway, the JAK-STAT signaling pathway, and cell adhesion molecules. The detailed GSEA results, including the top 50 enriched biological pathways, are presented in Supplementary Materials Table S2, ordered from highest to lowest on the basis of the NES. Subsequently, we screened the Hub genes, including Hla-a, B2m, CD4, CD8a, Jak1, Ifng, Klrk1, Cxcr4, Tnf, Akt1, Itgam, and Nfkb1, from these eight significant pathways using the STRING database and Cytoscape software (see Supplementary Materials Figure S1). Next, we performed qRT-PCR to validate these 12 genes.

Compared with that in the NC group, the expression of Hla-a, B2m, CD4, CD8a, Ifng, Klrk1, and Cxcr4 decreased, whereas the expression of Akt1, Nfkb1, Jak1, Tnf, and Itgam increased (Figure 4I,J). These findings suggest that USP18 may play a crucial role in CRC progression by modulating tumor immunity.

### 3.5. USP18 and Immune Cell Infiltration

Research reports have demonstrated that USP18 affects immune responses by regulating I-IFN-dependent signaling pathways [34,35,36]. We continued to investigate the link between USP18 expression and immune infiltration in CRC.

**Figure 5:**
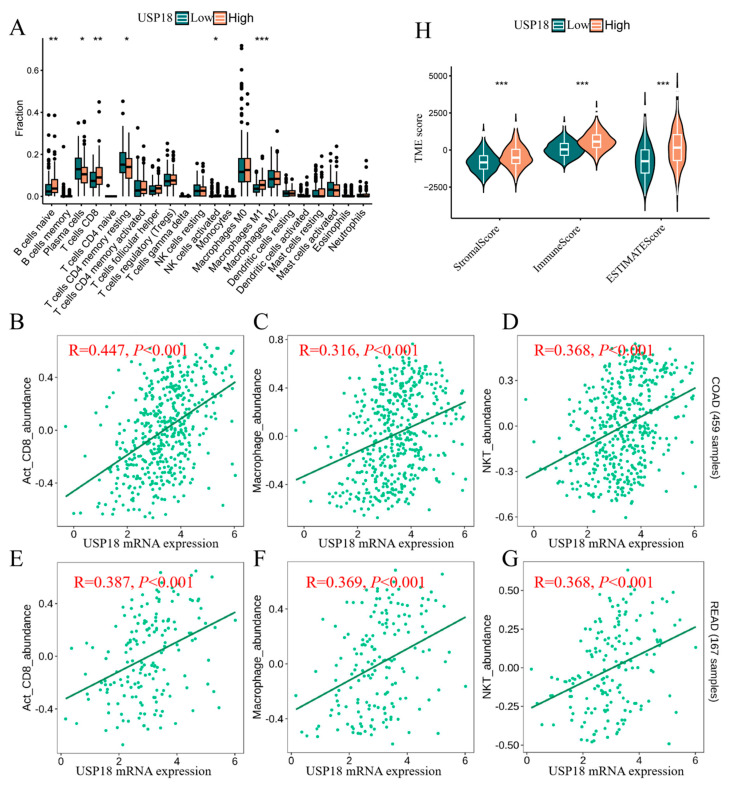
Correlation between USP18 and the tumor microenvironment. (**A**) Differences in the proportions of immune cells between the high-USP18 and low-USP18 groups. (**B**–**D**) Correlations between USP18 expression and the abundances of activated CD8+ T cells (B), activated dendritic cells (**C**), and NK T cells (**D**) in COAD (*n* = 459). (**E**–**G**) Correlations between USP18 expression and the abundances of activated CD8+ T cells (**E**), activated dendritic cells (**F**), and NK T cells (**G**) in READ patients (*n* = 167). (**H**) Comparison of TME scores between the high-USP18 and low-USP18 groups. The cell subpopulations were categorized into a high-USP18 group (orange) and a low-USP18 group (cyan) on the basis of USP18 expression levels. * *p*  <  0.05, ** *p*  <  0.01, *** *p*  <  0.001.

As shown in Figure 5A, patients with high USP18 expression had a greater proportion of antitumor immune cells, including activated natural killer cells (*p* < 0.05), M1-type macrophages (*p* < 0.001), and CD8-type T cells (*p* < 0.05). Tumor-infiltrating lymphocytes (TILs) are strongly associated with improved cancer prognosis [33]. Using the TISIDB tool, we explored the types of TILs that may be regulated by USP18 in CRC patients. The analysis indicated that USP18 expression was significantly positively correlated with natural killer T cells, activated CD8+ T cells, M1-type T cells, and M1-type macrophages (Figure 5B–G). We subsequently compared the differences in immune scores, stromal scores, and ESTIMATE scores between patients with high and low USP18 expression. As shown in Figure 5H, the immune score, stromal score, and ESTIMATE score were significantly greater in the USP18 high-expression group than in the low-expression group (*p* < 0.001). These findings suggest that USP18 may play an important role in the tumor microenvironment, particularly in increasing the abundance of immune components.

**Figure 6:**
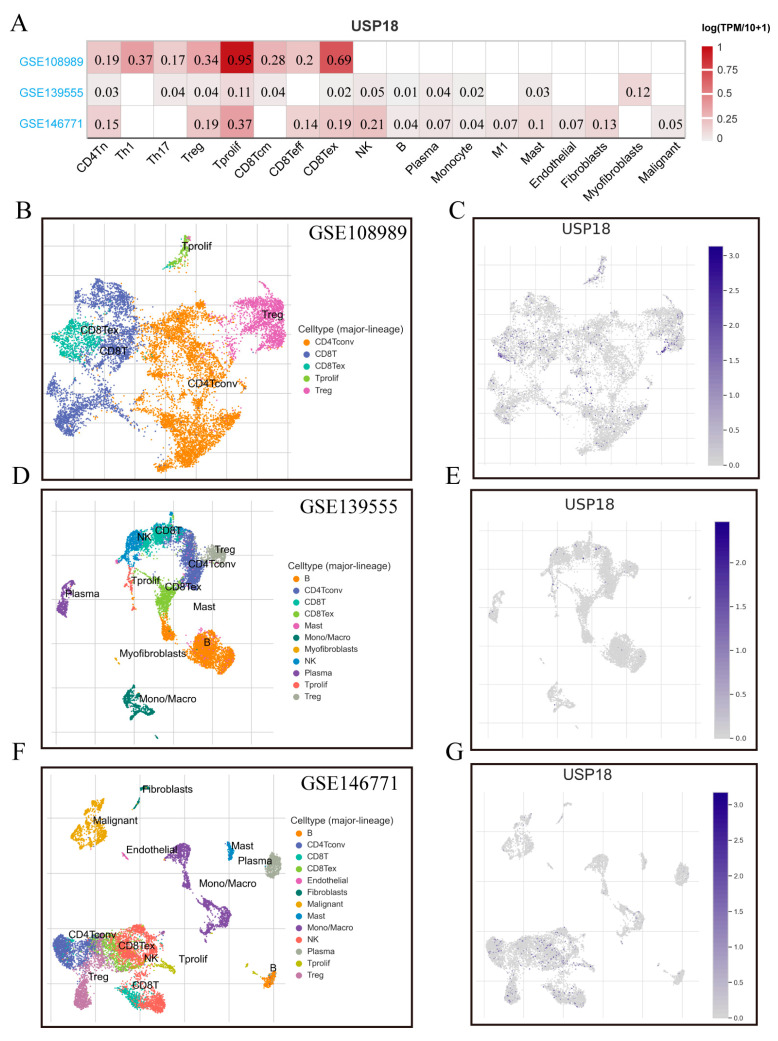
Correlation of USP18 with the TME in CRC according to the TISCH database. (**A**) Correlation of USP18 with the TME in CRC. (**B**,**D**,**F**) Annotation and distribution of immune cell types in the GSE108989 (*n* = 12), GSE139555 (*n* = 32), and GSE146771 (*n* = 20) datasets. (**C**,**E**,**G**) Proportion of USP18 among different immune cell types in the GSE108989, GSE139555, and GSE146771 datasets.

Using single-cell analysis technologies, the association between USP18 and the tumor microenvironment (TME) was investigated. As illustrated in Figure 6A, within the colorectal cancer datasets (GSE108989, GSE139555, and GSE146771), USP18 was predominantly expressed in immune cells, fibroblasts, and malignant cells, particularly in CD8+ T cells. Specifically, in the GSE108989 dataset, USP18 was highly expressed in proliferating T cells (Tprolif cells) and moderately expressed in regulatory T cells (Tregs), exhausted CD8+ T cells (CD8+ Tex cells), and Th1 cells. In the GSE139555 dataset, USP18 expression was primarily noted in malignant cells. Moreover, in the GSE146771 dataset, USP18 expression was increased in proliferating T cells and exhausted CD8+ T cells. The t-SNE plots (Figure 6B,D,F) display the distributions of major immune cell types across different datasets. The results shown in Figure 6C,E,G indicate a significant increase in USP18 expression in Tprolif and CD8+ Tex cells. USP18 may play a crucial role in Tprolif and the regulation of immune responses, explaining, to some extent, the improved prognosis in CRC patients.

### 3.6. Relationships of USP18 with Immune Checkpoint Genes in CRC

**Figure 7:**
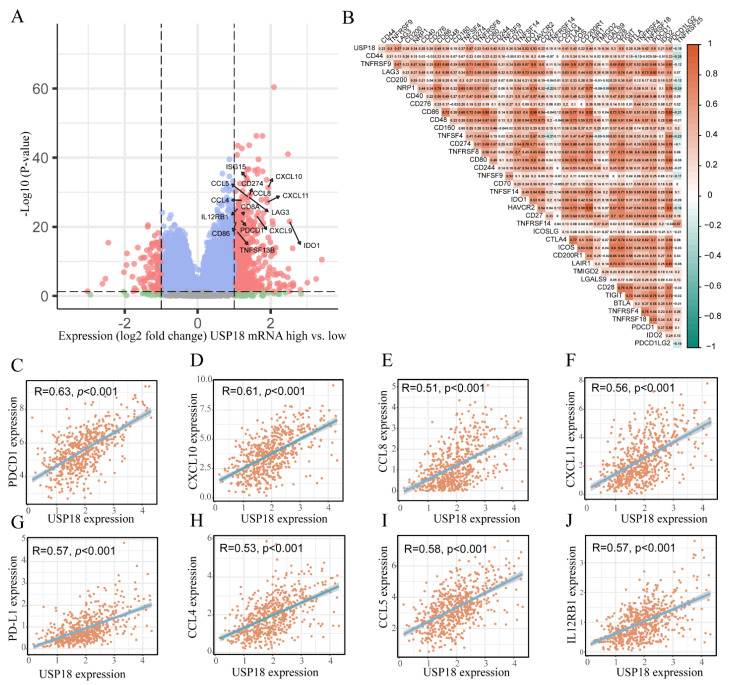
Association of USP18 with immune checkpoint genes. (**A**) Differential gene expression between the high USP18 expression group and the low USP18 expression group. (**B**) Correlations between USP18 and immune checkpoint inhibitory genes. (**C**–**H**) Correlations between USP18 and the immunomodulatory factors PDCD1 (**C**), CXCL10 (**D**), CCL8 (**E**), CXCL11 (**F**), PD-L1 (**G**), CCL4 (**H**), CCL5 (**I**), and IL12RB1 (**J**).

In further research, we explored the relationships between USP18 and immune checkpoint genes. CRC patients were divided into high- and low-expression groups on the basis of USP18 expression levels, and the DEGs between the two groups were screened. The results revealed that the high USP18 expression group was enriched with multiple immune activation-related genes, such as CD86, IL12RB1, CXCL10, ISG15, CXCL9, CCL4, CCL5, CD8A, PDCD1 (PD1), CD274 (PD-L1), and CCL8, which were significantly upregulated (Figure 7A). Additionally, USP18 was positively associated with several genes related to DC, T cell, and NK cell recruitment, including CCL4, CCL5, CCL8, CXCL11, CXCL10, and IL12RB1. Interestingly, we also found that the expression of immune checkpoint genes, including PD-L1 and PD-1, was positively correlated with USP18 expression (Figure 7C–J). On the basis of these findings, we performed a correlation analysis between USP18 and immune checkpoint gene sets. The results (Figure 7B) revealed that 37 immune checkpoint genes, including PD-L1, LAG3, PD1, IDO1, and TIGIT, were positively correlated with USP18 mRNA expression, further indicating the potential key role of USP18 in regulating the immune surveillance environment in CRC.

### 3.7. Regulation of PD-L1 Expression by USP18 Enhances Targeted Immunotherapy in CRC

**Figure 8:**
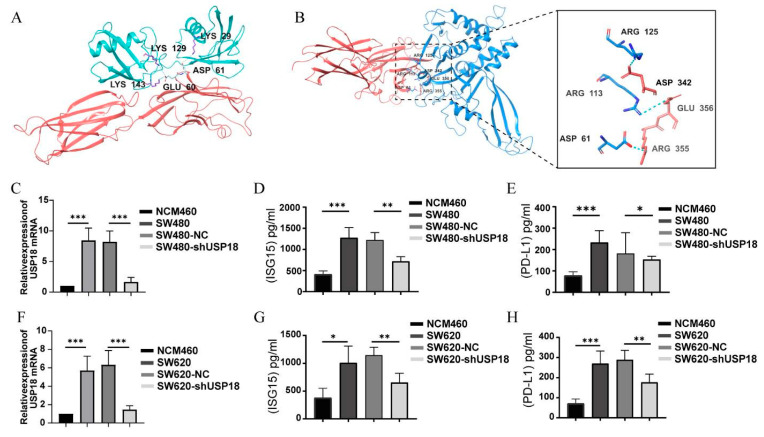
USP18 may regulate PD-L1 expression. (**A**) Molecular docking modeling between ISG15 and PD-L1 using HDOCK. Red indicates the PD-L1 protein, and cyan indicates the ISG15 protein. (**B**) Diagram of the molecular docking model between USP18 and PD-L1. Red indicates the PD-L1 protein, and blue indicates USP18. (**C**,**F**) Expression of USP18 mRNA in CRC. (**D**,**E**,**G**,**H**) The expression of ISG15 and PD-L1 in CRC supernatants was measured via an ELISA. Representative data were collected from at least three independent experiments and are expressed as the means ± SD. * *p* <  0.05, ** *p*  <  0.01, *** *p*  <  0.001.

Recent studies have shown that increased USP18 expression can affect the molecular stability of PD-L1 [36]. To preliminarily explore the impact of USP18 on the immune checkpoint blockade response, this study employed molecular docking analysis with HDOCK to assess the interactions between ISG15 and PD-L1, as well as between USP18 and PD-L1. As shown in Figure 8A,B, ISG15 and USP18 interact with PD-L1 to form stable complexes. We focused on the interactions among the amino acid residues ASP 61, ARG 113, ARG 125, ASP 342, GLU 356, and ARG 355, which primarily enhance the structural stability of the complexes through the formation of salt bridges and hydrogen bonds. The thermodynamic analysis presented in Table 1 reveals that the ISG15-PD-L1 complex has a binding free energy (ΔG) of −11.3 kcal/mol and a dissociation constant (Kd) of 4.9 × 10^−9^ M, demonstrating high affinity and significant binding stability. This finding is consistent with previous findings on ISG15-targeted glycosylation of PD-L1 [22]. Additionally, the USP18-PD-L1 complex exhibited a binding free energy of −12.0 kcal/mol and a dissociation constant of 1.6×10^−09^ M, further confirming the high stability of the complex. Moreover, an exhaustive analysis of amino acid residue contacts within both complexes was conducted, with particular attention to the number of contacts between charged polar residues and nonpolar residues, with the ISG15-PD-L1 and USP18-PD-L1 complexes having contact counts of 12 and 24 and 20 and 18, respectively. These data highlight the decisive role of electrostatic and hydrophobic interactions in maintaining the structural stability of the complexes. We subsequently used an ELISA to analyze the impact of USP18 on PD-L1 expression. The results, depicted in Figure 8C–H, show that silencing USP18 in CRC reduces the protein expression of ISG15 and PD-L1. Compared with those in the control group, the SW480/620 and SW480/620-NC groups presented significantly greater levels of ISG15 and PD-L1. On the basis of these findings, we propose that USP18 recruits T lymphocytes and that suppressing USP18 decreases PD-L1 levels in tumor cells. This reduction permits antitumor T cells to infiltrate the tumor microenvironment without suppressing their activity, thereby enhancing antitumor immunity.

## 4. Discussion

In recent studies, USP18 has been found to be highly expressed in many types of tumors and to play a significant role in tumorigenesis and progression [37], such as proliferation [38,39], pyroptosis [40], autophagy [41], immunity [42], and apoptosis [43,44]. As a principal DUB, USP18 is responsible for removing ISG15 from conjugated proteins. Previous studies have shown that the absence of USP18 increases ISGylation and, conversely, reduces cancer growth by regulating unstable growth modulators and inducing apoptosis [45,46,47]. In addition, Usp18 deficiency severely impairs cancer progression and improves survival [40]. Our research indicated that high levels of USP18 generally correlate with better outcomes in CRC patients and are closely linked to clinicopathological features. In this study, we demonstrated that silencing USP18 promotes CRC growth and proliferation, thus exerting a protumorigenic effect. These reports suggest that USP18 may exhibit both anticancer and procancer activities, depending on the target cells within the TME. The potential mechanisms underlying these differences may warrant further investigation.

Previous studies have indicated that inhibition of USP18 activity enhances the response to IFN therapy [34,48]. Notably, USP18 regulates the development of dendritic cells through its inhibitory effect on type I IFN signal transduction. These cells play crucial roles in linking innate and adaptive immune responses [35,49]. Androgen-activated androgen receptors upregulate the expression of USP18, suppress TAK1 phosphorylation, and inhibit NF-κB activation in antitumor T cells, thereby increasing the efficacy of PD-1 immune therapy [50]. Mammary epithelial cells lacking USP18 induce the recruitment of Th1-subtype CD4 T cells by increasing IFN-γ-mediated Cxcl10 secretion, thus forming a tumor-suppressive microenvironment [51].

In this study, we found that multiple immune-related pathways were significantly enriched in the USP18 high-expression group. We screened key genes in these pathways and verified by qRT-PCR that silencing USP18 resulted in decreased expression of Hla-a, B2m, CD4, CD8a, Ifng, Klrk1, and Cxcr4 and elevated expression of Akt1, Nfkb1, Jak1, Tnf, and Itgam, which supports its potential immune regulatory role. We also demonstrated that high expression of USP18 increased the number of activated NK cells, CD8+ T cells, and M1-type macrophages in CRC and that immunity scores, stromal scores, and ESTIMATE scores were significantly increased. Furthermore, single-cell sequencing revealed that USP18 was significantly expressed mainly in tumor-infiltrating T cell subsets, especially in CD8+ T cells. This finding is consistent with previous conclusions [40,48]. Our results reveal the potential mechanism by which USP18 regulates the TME in CRC, suggesting that USP18 may broadly impact overall prognosis by promoting the aggregation of stromal and immune cells in patients.

Chemokines play crucial roles in directing immune cell migration, which is required for the initiation and delivery of an effective antitumor immune response [52]. Chemokines play crucial roles in recruiting and activating antitumor immune cells, especially NKs, DCs, and T cells, in the TME. For example, CXCL9 and CXCL10 are two important chemokines that are involved in antitumor immunity by binding to their receptor, CXCR3, which promotes the migration of T cells and NK cells into the tumor microenvironment [53,54,55]. The expression of USP18 in CRC is positively related to the expression of chemokines such as CCL4, CCL5, CCL8, CXCL10, CXCL11, and IL12RB1. In addition, we also found that the expression of immunosuppressive molecules, including PD-L1, PD-1, LAG3, TNFSF13B and IDO1, was enriched and positively correlated with high USP18 expression, which further suggested that USP18 plays an important role in regulating antitumor immunity in CRC.

In recent years, immunotherapies such as PD-1/PD-L1-targeted therapies and chimeric antigen receptor (CAR) T cell therapies have proven to be effective treatments in clinical oncology [50,56]; however, how USP18 coordinates PD-L1 protein levels in response to tumor microenvironmental signals in CRC remains unknown. Based on these findings, we used molecular docking simulations to show that USP18 and ISG15 can bind to PD-L1. This study presents the first molecular 3D model of USP18 and ISG15 coupling to PD-L1 in CRC. We subsequently analyzed the expression levels of ISG15 and PD-L1 and discovered that suppression of USP18 reduced the levels of both ISG15 and PD-L1. This finding is consistent with the conclusions of Zheng Rui et al., who reported that USP18 downregulation can reduce the protein stability of PD-L1 and weaken the PD-L1/PD-1 interaction, thereby enhancing the antitumor immune response and ultimately inhibiting bladder tumorigenesis [57].

This study has certain limitations, as the molecular mechanisms by which USP18 regulates PD-L1 expression and whether there is a direct interaction remain to be elucidated. These issues need to be addressed through further in vivo and in vitro experimental studies.

## 5. Conclusions

In brief, our study demonstrated that high USP18 expression has good prognostic value and strong potential to enhance tumor-specific immunity in CRC patients. Silencing USP18 can promote the growth and proliferation of CRC. Additionally, USP18 may target PD-L1 through ISG15 and inhibit the growth of CRC in vivo, suggesting a potential strategy for targeting PD-L1-mediated immune escape in tumor cells. These findings reveal the complex role of USP18 in CRC and provide new insights into how it influences disease progression by modulating the tumor immune microenvironment. These findings have significant implications for future research and clinical applications, with the potential to advance the development of precision medicine and ultimately improve treatment outcomes and prognoses for CRC patients.

## Figures and Tables

**Table 1:** Binding characteristics and high affinity of the USP18-PD-L1 and ISG15-PD-L1 complexes.

Protein-Protein Complex	Docking Score	ΔG (kcal mol^−1^)	Kd (M)	ICs Charged–Charged	ICs Charged–Apolar
USP18-PD-L1	−256.35	−11.3	4.9 × 10^−9^	12	24
ISG15-PD-L1	−223.24	−12.0	1.6 × 10^−9^	20	18

## Data Availability

The data presented in this study are available upon request from the corresponding author.

## Supplementary Materials

The following supporting information can be downloaded at: https://www.mdpi.com/article/10.3390/biom14091191/s1, Figure S1: Protein-protein interaction (PPI) network analysis of significantly enriched pathways in the USP18 high-expression group; Table S1: Primers used for analysis in this study; Table S2: Top 50 pathways Enriched by GSEA Analysis; Figure S2: Original Images for Blots.
