# Supplementary material for: USP18 Is Associated with PD-L1 Antitumor Immunity and Improved Prognosis in Colorectal Cancer
Source: Biomolecules. 2024 Sep 21;14(9):1191. doi: 10.3390/biom14091191 (PMC11430364; doi:10.3390/biom14091191)

Supplementary Figure S1

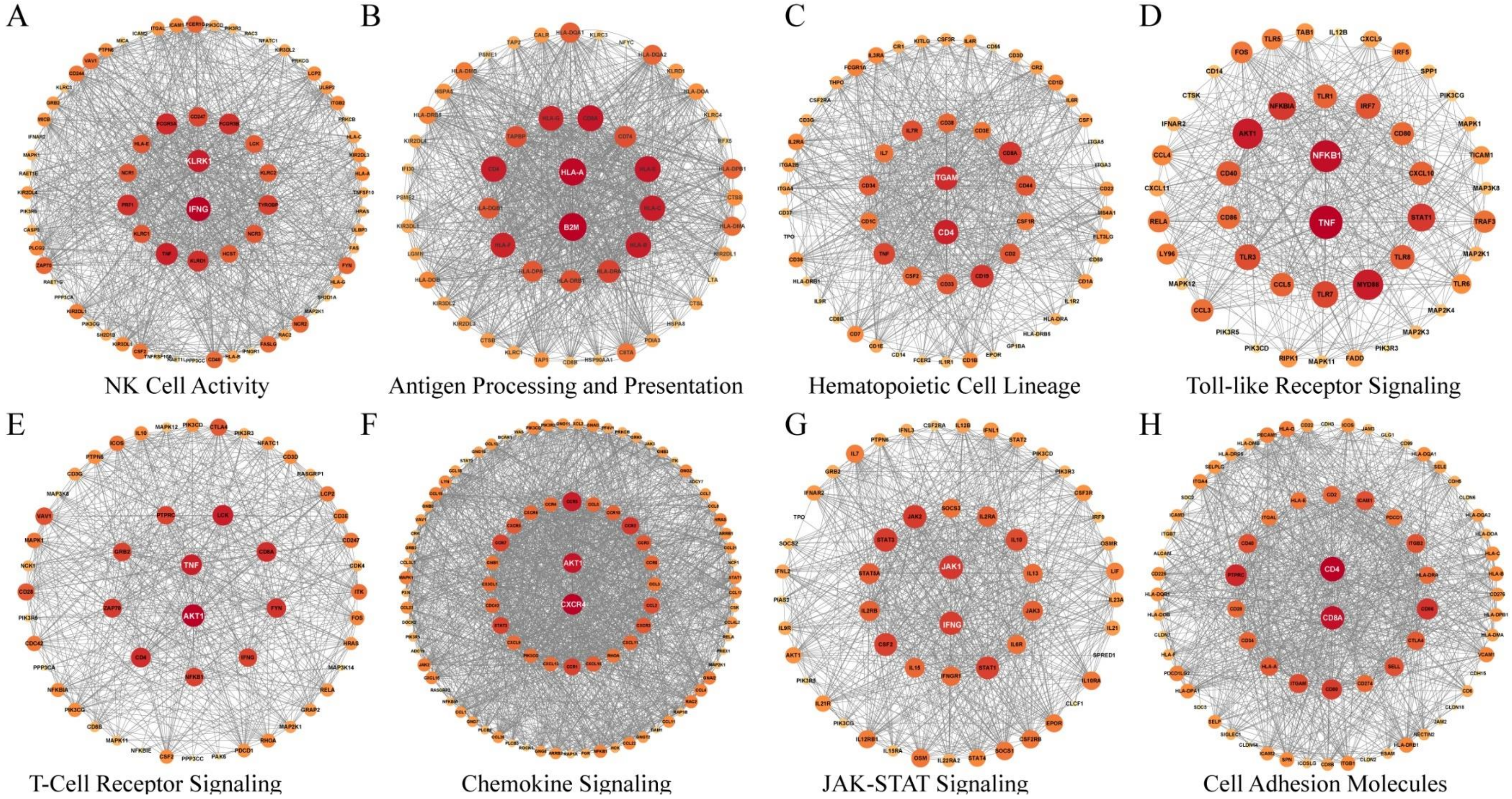

Supplementary Figure S1. Protein-protein interaction (PPI) network analysis of significantly enriched pathways in the USP18 high-expression group. Each panel (A-H) illustrates the PPI networks of core genes within eight significantly enriched biological pathways: **(A)** NK Cell Activity; **(B)** Antigen Processing and Presentation; **(C)** Hematopoietic Cell Lineage; **(D)** Toll-like Receptor Signaling; **(E)** T-Cell Receptor Signaling; **(F)** Chemokine Signaling; **(G)** JAK-STAT Signaling; and **(H)** Cell Adhesion Molecules. Red nodes represent hub genes with high connectivity within the network, and the size of each circle indicates the degree of connectivity, highlighting their potential roles as key regulators in their respective pathways. The PPI networks were constructed using the STRING database and visualized with Cytoscape software.

Supplementary Table S1

Primers used for analysis in this study

Gene name	Forward primer (5'-3')	Reverse primer (5'-3')
Usp18	TGGACAGACCTCRCTCRCCTT	CTGTCCTCRCATCTTCTCCACRCA
Akt1	GGACTACTTGCACTCCGAGAAG	CATAGTGGCACCGTCCTTGATC
B2m	CCACTGAAAAAGATGAGTATGCCT	CCAATCCAAATGCGGCATCTTCA
Cd4	CCTCCTGCTTTTCATTGGGCTAG	TGAGGACACTGGCAGGTCTTCT
Cd8a	ACTTGTGGGGTCCTTCTCCTGT	TGTCTCCCGATTTGACCACAGG
Cxcr4	CTCCTCTTTGTCATCACGCTTCC	GGATGAGGACACTGCTGTAGAG
Hla-a	AGATACACCTGCCATGTGCAGC	GATCACAGCTCCAAGGAGAACC
Ifng	GAGTGTGGAGACCATCAAGGAAG	TGCTTTGCGTTGGACATTCAAGTC
Klrk1	GGTATGAGAGCCAGGCTTCTTG	GAATGGAGCCATCTTCCCACTG
Itgam	GGAACGCCATTGTCTGCTTTCG	ATGCTGAGGTCATCCTGGCAGA
Jak1	GAGACAGGTCTCCCACAAACAC	GTGGTAAGGACATCGCTTTTCCG
Nfkb1	GCAGCACTACTTCTTGACCACC	TCTGCTCCTGAGCATTGACGTC
Tnf	CTCTTCTGCCTGCTGCACTTTG	ATGGGCTACAGGCTTGTCACTC
β-actin	CACCATTGCRCAATGACRCGGTTC	AGGTCTTTCRCGGATGTCCACGT

Supplementary Table S2

Top 50 Pathways Enriched by GSEA Analysis

NAME	SIZE	ES	NES	NOM p-val	FWER p-val	FDR q-val	RANK AT MAX	LEADING EDGE
KEGG_NATURAL_KILLER_CELL_MEDIATED_CYTOTOXICITY	132	0.71085864	2.6227784	0	0.001	0	5352	tags=56%, list=10%, signal=62%
KEGG_VIRAL_MYOCARDITIS	68	0.7681612	2.5088036	0	0.001	0	3696	tags=53%, list=7%, signal=57%
KEGG_ANTIGEN_PROCESSING_AND_PRESENTATION	81	0.81113404	2.50676	0	0.001	0	4254	tags=64%, list=8%, signal=69%
KEGG_HEMATOPOIETIC_CELL_LINEAGE	85	0.77850634	2.3939984	0	0.001	0	7328	tags=68%, list=13%, signal=79%
KEGG_TOLL_LIKE_RECEPTOR_SIGNALING_PATHWAY	102	0.6389846	2.3826756	0	0.001	0	6201	tags=46%, list=11%, signal=52%
KEGG_CYTOKINE_CYTOKINE_RECEPTOR_INTERACTION	264	0.640456	2.3559756	0	0.002	4.79E-04	6217	tags=47%, list=11%, signal=53%
KEGG_SYSTEMIC_LUPUS_ERYTHEMATOSUS	55	0.8280378	2.3424969	0	0.002	4.10E-04	3198	tags=62%, list=6%, signal=66%
KEGG_AUTOIMMUNE_THYROID_DISEASE	50	0.79583764	2.3339994	0	0.002	4.93E-04	3198	tags=56%, list=6%, signal=59%
KEGG_LEISHMANIA_INFECTION	70	0.7785704	2.3245568	0	0.002	6.77E-04	5683	tags=66%, list=10%, signal=73%
KEGG_CHEMOKINE_SIGNALING_PATHWAY	188	0.6331848	2.3107755	0	0.003	7.26E-04	6348	tags=51%, list=11%, signal=57%
KEGG_INTESTINAL_IMMUNE_	46	0.82850665	2.2838182	0	0.005	0.0013	6651	tags=74%, list=12%, signal=84%

NETWORK_FOR_IGA_PRODUCTION						74282		
KEGG_CELL_ADHESION_MOLECULES_CAMS	131	0.70214033	2.2608757	0	0.007	0.0016 35042	6769	tags=56%, list=12%, signal=64%
KEGG_TYPE_I_DIABETES_MELLITUS	41	0.86004084	2.2590268	0	0.007	0.0015 0927	3198	tags=68%, list=6%, signal=72%
KEGG_APOPTOSIS	87	0.5956639	2.2506852	0	0.01	0.0017 80696	7117	tags=46%, list=13%, signal=53%
KEGG_GRAFT_VERSUS_HOST_DISEASE	37	0.9262072	2.2165234	0	0.012	0.0022 54067	3198	tags=86%, list=6%, signal=92%
KEGG_ASTHMA	28	0.8511952	2.194355	0	0.013	0.0023 19616	1928	tags=57%, list=3%, signal=59%
KEGG_PRION_DISEASES	35	0.6652095	2.1773286	0	0.014	0.0023 24386	9858	tags=51%, list=18%, signal=63%
KEGG_ALLOGRAFT_REJECTION	35	0.89588344	2.158577	0	0.014	0.0026 87617	3198	tags=80%, list=6%, signal=85%
KEGG_CYTOSOLIC_DNA_SENSING_PATHWAY	54	0.5938175	2.1526842	0	0.016	0.0027 4611	4072	tags=35%, list=7%, signal=38%
KEGG_AMINO_SUGAR_AND_NUCLEOTIDE_SUGAR_METABOLISM	43	0.6566491	2.1321065	0.0019 34236	0.025	0.0036 43385	7208	tags=58%, list=13%, signal=67%
KEGG_GALACTOSE_METABOLISM	26	0.70172143	2.1277835	0	0.028	0.0037 49759	7846	tags=58%, list=14%, signal=67%
KEGG_JAK_STAT_SIGNALING_PATHWAY	155	0.53392535	2.1134198	0	0.032	0.0041 54943	5330	tags=34%, list=10%, signal=38%
KEGG_PATHOGENIC_ESCHERI	56	0.60393894	2.1133766	0	0.032	0.0039	7277	tags=52%, list=13%, signal=60%

CHIA_COLI_INFECTION						74293		
KEGG_B_CELL_RECEPTOR_SIGNALING_PATHWAY	75	0.6006643	2.107926	0.002	0.034	0.004192148	7117	tags=56%, list=13%, signal=64%
KEGG_AMYOTROPHIC_LATER	53	0.55418897	2.093562	0	0.04	0.0046	6867	tags=43%, list=12%, signal=50%
AL_SCLEROSIS_ALS						06839		
KEGG_RIG_I_LIKE_RECEPTOR_SIGNALING_PATHWAY	70	0.5483941	2.0782495	0	0.043	0.004809994	1975	tags=24%, list=4%, signal=25%
KEGG_T_CELL_RECEPTOR_SIGNALING_PATHWAY	108	0.56599784	2.0709586	0.004065041	0.045	0.004995836	5822	tags=48%, list=11%, signal=54%
KEGG_FC_GAMMA_R_MEDIATED_PHAGOCYTOSIS	96	0.5597831	2.0564995	0.002024292	0.05	0.005355535	5599	tags=44%, list=10%, signal=49%
KEGG_REGULATION_OF_ACTIN_CYTOSKELETON	213	0.51076216	2.0560532	0.003937008	0.051	0.005209388	6128	tags=40%, list=11%, signal=45%
KEGG_PRIMARY_IMMUNODEFICIENCY	35	0.75839436	2.052893	0	0.052	0.005144933	6308	tags=71%, list=11%, signal=81%
KEGG_PANCREATIC_CANCER	70	0.5521442	2.0309465	0.00203666	0.065	0.006347163	5673	tags=39%, list=10%, signal=43%
KEGG_LEUKOCYTE_TRANSENDOTHELIAL_MIGRATION	116	0.561556	2.0291739	0.001964637	0.066	0.006340498	6769	tags=52%, list=12%, signal=59%
KEGG_NOD_LIKE_RECEPTOR_SIGNALING_PATHWAY	62	0.55880487	1.9893743	0.007858546	0.096	0.009448195	7441	tags=50%, list=13%, signal=58%
KEGG_GLYCOSAMINOGLYCAN_BIOSYNTHESIS_KERATAN_SULFATE	15	0.79026735	1.9889854	0	0.096	0.009170308	6961	tags=67%, list=13%, signal=76%
KEGG_LYSOSOME	121	0.5959955	1.9875457	0.003802281	0.096	0.008971777	6403	tags=47%, list=12%, signal=53%

KEGG_FC_EPSILON_RI_SIGNALING_PATHWAY	79	0.50391376	1.985678	0.004056795	0.097	0.008848486	5165	tags=39%, list=9%, signal=43%
KEGG_RENAL_CELL_CARCINOMA	70	0.5270792	1.9762067	0.001980198	0.103	0.009340053	5568	tags=40%, list=10%, signal=44%
KEGG_FRUCTOSE_AND_MANNOSE_METABOLISM	33	0.65734243	1.9737165	0.003846154	0.104	0.009196828	4787	tags=45%, list=9%, signal=50%
KEGG_SPHINGOLIPID_METABOLISM	39	0.5763436	1.9690338	0.003787879	0.11	0.009404861	4817	tags=41%, list=9%, signal=45%
KEGG_VEGF_SIGNALING_PATHWAY	76	0.49845186	1.9649024	0.00409836	0.118	0.009696019	5236	tags=38%, list=9%, signal=42%
KEGG_PATHWAYS_IN_CANCER	325	0.46548432	1.9528705	0.004081633	0.13	0.010430144	7389	tags=39%, list=13%, signal=45%
KEGG_TYPE_II_DIABETES_MELLITUS	47	0.5465346	1.943221	0.001964637	0.142	0.011486929	5914	tags=38%, list=11%, signal=43%
KEGG_ENDOCYTOSIS	181	0.45274866	1.899094	0.005882353	0.192	0.017925749	5278	tags=33%, list=10%, signal=36%
KEGG_FOCAL_ADHESION	199	0.53741384	1.8957721	0.010245902	0.194	0.017793063	6135	tags=43%, list=11%, signal=48%
KEGG_COMPLEMENT_AND_COAGULATION_CASCADES	69	0.58149475	1.8913141	0.014583333	0.2	0.018136814	7093	tags=55%, list=13%, signal=63%
KEGG_MAPK_SIGNALING_PATHWAY	267	0.43756336	1.8771985	0.006036217	0.213	0.020153377	7248	tags=37%, list=13%, signal=43%
KEGG_ECM_RECEPTOR_INTERACTION	84	0.6389261	1.8715124	0.011952192	0.224	0.020809116	7403	tags=55%, list=13%, signal=63%
KEGG_P53_SIGNALING_PATHWAY	68	0.5027761	1.8648314	0.005847953	0.232	0.0215031	7362	tags=44%, list=13%, signal=51%

KEGG_ACUTE_MYELOID_LEUKEMIA	57	0.5207719	1.8616832	0.007874016	0.234	0.021482015	7150	tags=40%, list=13%, signal=46%
KEGG_GLYCOLYSIS_GLUCONEOGENESIS	62	0.5826926	1.8558997	0.005847953	0.238	0.021793097	4883	tags=37%, list=9%, signal=41%

Supplementary Figure S2

Full uncropped gel image for Fig.1D

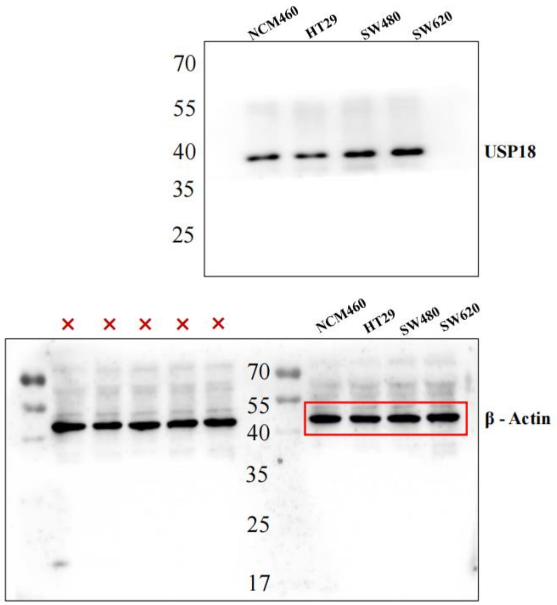

Full uncropped gel image for Fig.3B

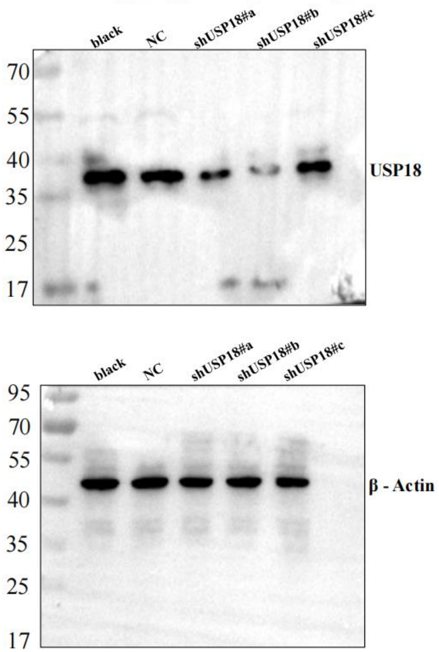

Full uncropped gel image for Fig.3D

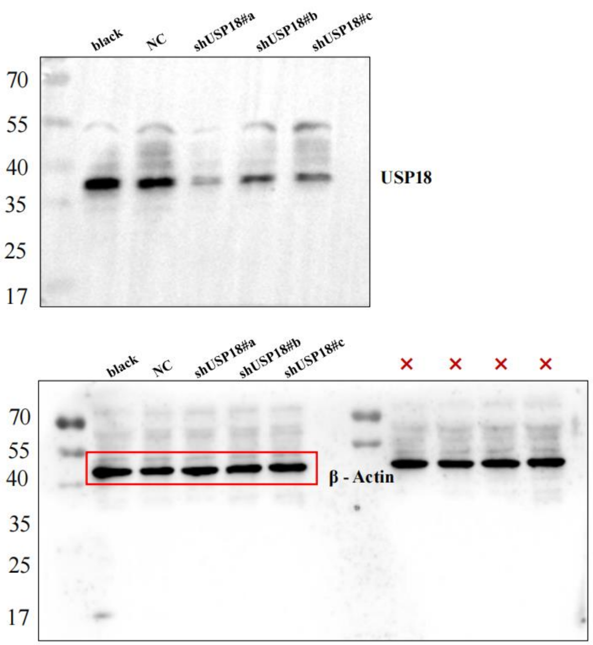

Supplement: Supplementary file 1 [file biomolecules-14-01191-s001.zip › biomolecules-3148477-supplementary.pdf]
